# Management of Pulmonary Arterial Hypertension in Pregnancy: Experience from a Nationally Accredited Center

**DOI:** 10.3390/jcdd9060195

**Published:** 2022-06-18

**Authors:** Anjali Vaidya, Estefania Oliveros, Wadia Mulla, Diana Feinstein, Laura Hart, Paul Forfia

**Affiliations:** 1 Pulmonary Hypertension, Right Heart Failure, CTEPH Program, Temple Heart and Vascular Institute, Department of Medicine, Temple University Hospital, Philadelphia, PA 19197, USA; estefania.oliverossoles@tuhs.temple.edu (E.O.); paul.forfia@tuhs.temple.edu (P.F.); 2 Division of Maternal Fetal Medicine, Department of Obstetrics, Gynecology, and Reproductive Sciences, Temple University Hospital, Philadelphia, PA 19197, USA; wadia.mulla@tuhs.temple.edu (W.M.); laura.hart@tuhs.temple.edu (L.H.); 3 Obstetric Anesthesiology, Department of Anesthesiology, Temple University Hospital, Philadelphia, PA 19197, USA; diana.feinstein@tuhs.temple.edu

**Keywords:** pulmonary hypertension, pregnancy, right ventricular dysfunction, right heart failure, maternal fetal medicine, cardio-obstetrics

## Abstract

(1) Background: In pulmonary arterial hypertension (PAH), pregnancy is regarded a contraindication due to high maternal and fetal morbidity and mortality. We report our experience in the management of pregnancies in PAH. (2) Methods: retrospective observational study in a nationally accredited pulmonary hypertension (PH) center from 2013 to 2021. (3) Results: seven pregnancies in six women with PAH, ranging from low to high risk and 21 to 37 years old. Half had known pre-existing PAH before pregnancy. One had a multifetal gestation, and one was pregnant twice under our care. PH medical therapy and serial clinical assessment throughout pregnancy were implemented with focused attention on optimizing right heart function. Delivery was planned by a multidisciplinary team involving PH cardiology, maternal fetal medicine, and obstetric anesthesiology. Patients delivered between 31 and 40 weeks of gestation; five of the seven were via cesarean section. All received regional anesthesia and were monitored in the PH intermediate step-down unit after delivery until discharge. In all cases, delivery was without complications with excellent outcomes for the mother and child. (4) Conclusions: Multidisciplinary and tailored management of PAH in pregnancy, emphasizing optimized right heart function prior to delivery, can result in excellent clinical outcomes in a referral PH center.

## 1. Introduction

Pulmonary arterial hypertension (PAH) is hemodynamically defined by the presence of pulmonary hypertension (PH) (mean pulmonary artery pressure greater than 20 mmHg), a pulmonary arterial wedge pressure ≤15 mmHg, and pulmonary vascular resistance (PVR) ≥3 mmHg/L/min. PAH carries high morbidity and mortality during pregnancy, with mortality rates reported as high as 30–50% [1,2,3,4,5,6]. Prognosis has improved in recent years given the introduction of new PAH medications in the field, but it remains one of the highest risk medical conditions for both mother and baby; in fact, according to guidelines, pregnancy is contraindicated in patients with PAH [7,8]. The World Health Organization (WHO) classifies PH as a class IV heart disease and advises against pregnancy. Fetal complications include miscarriage (5.6%), fetal loss (2%), preterm delivery (21.7%), fetal growth restriction (19%) and neonatal mortality (0.7%) [2]. Despite these risks, pregnancy does occur in PAH. 

PH management during pregnancy, as with the management of other cardiovascular disorders in pregnancy, should be aimed at optimizing the central pathophysiology of the condition and providing the greatest degree of achievable physiologic reserve in anticipation of the physiologic stresses inherent to pregnancy, labor, and delivery. The aim of this case series is to show our best practices when managing PAH patients in pregnancy. 

## 2. Materials and Methods

We retrospectively reviewed the medical records of patients with PAH seen in our tertiary care center. This is a single-center, retrospective study. The physicians in our program established our current PH center in 2013; data were collected on all patients since that time with pregnancy and PAH. Patients that carried pregnancy to delivery are discussed in detail as the primary objective of this study. Those that underwent medical termination of pregnancy are also described. We collected personal and family history, physical examination, laboratory assessment, cardiac evaluation (electrocardiogram, echocardiogram, right heart catheterization) and maternal and neonatal outcomes. The study protocol conforms to ethical guidelines of the 1975 Declaration of Helsinki as reflected in a prior approval by the IRB of our institution. 

Clinical assessment and risk stratification at the time of pregnancy involved a comprehensive assessment of clinical parameters typically used for PAH risk assessment. This included evidence for clinical right heart failure by jugular venous pressure (JVP), B-type natriuretic peptide (BNP), and functional status assessments including functional class and 6-min walk distance (6MWD). Echocardiography was performed at the beginning of pregnancy and was serially reassessed during pregnancy, with a focus on right heart function (RV systolic function, degree of tricuspid regurgitation (TR)) and other echo-Doppler parameters relevant to right heart-pulmonary vascular coupling, including the degree of systolic interventricular septal flattening and the presence and degree of ‘notching’ in the right ventricular outflow tract pulse wave Doppler profile [9]. Finally, if not carried out prior to pregnancy, right heart catheterization was performed.

The primary objectives of PH medical management (PH specific medical therapy and diuretics) before (when applicable) and during pregnancy in each patient were to improve right heart function as much as possible, with the goal of optimizing RV systolic function at the least degree of TR. The PH medical regimen for each individual patient and pregnancy was chosen based on what was deemed necessary to achieve normal or near normal RV function with mild or less TR, as opposed to applying a singular therapeutic approach for all patients. An important and related goal in this approach was to achieve a normal right atrial pressure, with the intent being to provide the patient with the greatest degree of cardiopulmonary reserve possible given the anticipated physiologic stress of progression through the third trimester and at the time of labor and delivery. 

PH medical therapy was modified if warranted at the time of pregnancy based on teratogenic effects and known safety profiles in PAH. Based on serial clinical and echo-Doppler assessment, PH medications and diuretics were added or titrated to achieve the optimal right heart function possible over the course of the pregnancy. The medication regimen and dosing varied depending on the individual need to achieve this. 

A multidisciplinary discussion involving PH cardiology, maternal fetal medicine (MFM), obstetrics anesthesia, labor and delivery nursing, pharmacy and, if warranted, pediatrics-neonatology, occurred early in the third trimester to establish the delivery plan. This included anticipated vaginal delivery versus cesarean section (c-section) and location of the planned delivery within the hospital setting. 

## 3. Results

We report on ten pregnancies with PAH at our institution between 2013 and 2021. This is a nationally accredited Pulmonary Hypertension Association PH Center for Comprehensive Care, including hundreds of female patients of child-bearing age during this period. Seven pregnancies in six patients were carried to delivery. Three pregnancies in three patients were terminated during the first trimester and will be discussed separately. The age of patients at the time of pregnancy ranged from 21 to 37 years old. Two of six patients (Patients 3 and 6) were diagnosed with PAH during their first pregnancy. All required PH medical therapy during pregnancy. Four patients were followed by our team prior to pregnancy; three pregnancies (Patient 1 and Patient 5) were planned and required PH medication adjustments at least 3 months prior to pregnancy. 

Clinical characteristics including PAH etiology, comorbid conditions, obstetric history, PAH medical therapy, timing and mode of delivery, anesthesia at time of delivery, postpartum length of stay, maternal and neonatal outcomes and postpartum mode of contraception are summarized in Table 1. All patients who underwent c-section had an obstetric indication, such as severe pre-eclampsia or prior c-section. 

Patient functional assessment and BNP level at baseline and at third trimester, as well as JVP at baseline and at the time of delivery are summarized in Table 2. The NYHA FC ranged from I-IV, with the two patients (Patient 6 and Patient 3) presenting with a new PAH diagnosis, on no PAH medical therapy, being FC III and IV at baseline. The 6MWD was not obtainable in Patient 2 (BMI 56) and Patient 3 (hospitalized with severe right heart failure), and otherwise ranged from 292 to 585 m in the remaining patients. Six of the seven patients had a BNP level <100 pg/mL at the time of delivery. All patients had a JVP ≤10 cm water at the time of delivery. 

Patient 1 and Patient 5 warranted discontinuation of ambrisentan prior to pregnancy due to teratogenic risk. Patient 1 remained on inhaled treprostinil therapy during both pregnancies. In her first pregnancy, BNP fell, and there was no significant change in RV function. However, during her second pregnancy, RV size, function, and TR worsened in the last week before delivery. Patient 5 continued her sildenafil therapy in pregnancy, with the addition of inhaled treprostinil. She maintained normal BNP, mild RV dilation and dysfunction, mild systolic septal flattening, and TAPSE 1.8 cm throughout pregnancy. 

Table 3 summarizes the echocardiographic variables of the patients immediately before or first obtained in pregnancy. Four of the six patients were treated with PAH medical therapy before pregnancy and two patients presented in the early third trimester with a new diagnosis of PAH, and thus were untreated at the time of presentation. Right ventricular cavity size and function as well as TR ranged from normal to severely abnormal. As expected, patients not on PAH medical therapy at the time of presentation had more severe RV dilatation, dysfunction, TR, and had evidence of mid-systolic notching of the RVOT Doppler signal (associated with a higher degree of PVR). In contrast, patients on PAH medical therapy before pregnancy had a less severe right heart dysfunction phenotype, with normal to mild RV dysfunction and evidence of a lower PVR, given either no notching or a late-notch pattern of the RVOT Doppler signal. 

**Figure 1:**
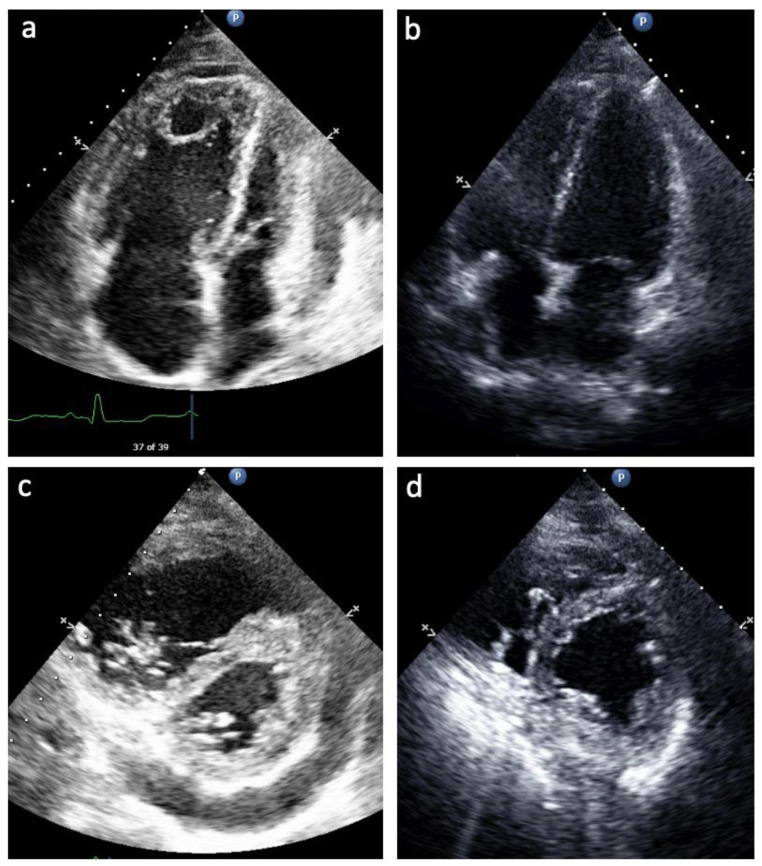
Echocardiogram from Patient 3 prior to (**a**,**b**) and after (**c**,**d**) PAH medication optimization. (**a**) Apical 4 Chamber view of enlarged RA and enlarged and hypertrophied RV with small and underfilled LV, LA; (**c**) Apical 4 Chamber view of normalized RA and RV size and function on PAH therapy; (**b**) Parasternal short axis view of severe systolic septal flattening, RV enlargement and hypertrophy, and pericardial effusion; (**d**) Parasternal short axis view of resolution of systolic septal flattening and pericardial effusion with smaller RV size.

Table 4 summarizes the echocardiographic variables of the patients just before delivery, at an average of 34 weeks’ gestation. In six of the seven pregnancies, patients had either mild RV systolic dysfunction or normal RV systolic function as well as either mild or no TR. In six of the seven pregnancies, there was either no RVOT Doppler notching or only late Doppler nothing, consistent with a lower PVR. No patients during any of the pregnancies had a pericardial effusion at the time of delivery. As discussed above, Patient 3 presented initially at 28 weeks with severe RV dysfunction and clinical right heart failure. In the setting of PAH medical therapy titration through the remainder of her pregnancy, her RV dilation and dysfunction improved from severe to mild, TAPSE from 1.3 cm to 1.8 cm, septal flattening from severe to mild, TR from severe to none, and RVOT Doppler notching from mid notch pattern to no evidence of RVOT Doppler notching (Figure 1). Patient 6 had an improvement in RV size from moderate to mildly enlarged, with resolution of systolic septal flattening and RVOT Doppler notch with PAH therapy by the third trimester. Patient 1, on her second pregnancy, did experience worsening RV size, function, and worsening TR on her echocardiogram in the last week before delivery. While she had no clinical right heart failure before hospital discharge postpartum, in the two weeks thereafter, she demonstrated elevated JVP and lower extremity edema. Given her worsening right heart function and clinical right heart failure, we counseled the patient away from breast feeding to resume therapy with her endothelin receptor antagonist, in addition to her other PAH medical therapies. 

Invasive hemodynamics were obtained in each patient (Table 5). In Patients 1 and 5, this was completed prior to pregnancy, whereas in the rest, this was carried out after pregnancy was already established to assess risk and guide medical therapy. No patients were diagnosed or treated solely on the basis of echocardiogram and noninvasive clinical assessment.

Formal risk calculation was performed using ESC/ERS (European Society of Cardiology/European Respiratory Society) 2015 Guidelines for each patient at baseline or early in pregnancy, and again during the third trimester (Table 6). Initially, three of the patients had PAH with moderate or high risk assessment. With initiation and titration of PAH medical therapy in all three, they improved to low risk by the third trimester and time of delivery. All other patients maintained low risk throughout pregnancy to the time of delivery. 

The timing of delivery was individualized considering maternal and fetal conditions and the severity and optimization of PAH. There was variation in the delivery mode between vaginal deliveries (three of seven) and c-section deliveries (four of seven). All deliveries occurred either on the Labor and Delivery floor or in the Obstetrics operating room (OR). Intravenous (IV) fluids were used sparingly (with colloids preferred over crystalloids), and IV medications were concentrated when able to minimize volume loading and the risk of right heart failure. Central venous monitoring was not used at the time of delivery, given each patient had a reliable JVP, which was monitored serially in lieu of invasive monitoring. Pulmonary artery (PA) catheters were not used at the time of delivery. Arterial lines were used at the time of c-section for systemic blood pressure monitoring in anticipation of the need for systemic vasopressor support.

Two of the patients, Patient 2 and Patient 5, developed pre-eclampsia in the third trimester. Patient 2 was diagnosed with gestational hypertension at 30 weeks and received corticosteroids for fetal lung maturity. She was diagnosed with pre-eclampsia with severe features at 30 weeks 6 days and underwent expectant management until 31 weeks 2 days when she was delivered due to worsening transaminitis. She had multiple pre-eclampsia risk factors, including nulliparity, morbid obesity (pre-pregnancy BMI 56), obstructive sleep apnea, chronic hypoxemia, and multiple gestation. Patient 5 was diagnosed with pre-eclampsia with severe features at 33 weeks, received corticosteroids, underwent expectant management and was delivered at 33 weeks 3 days due to worsening thrombocytopenia. Her risk factors for pre-eclampsia included nulliparity and chronic hypoxemia. Both patients received magnesium sulfate for 24 hours (h) postpartum for seizure prophylaxis. 

After delivery, once bleeding or any obstetric complications were ruled out, the patients were transferred to the PH cardiology inpatient service for ongoing monitoring and management until discharge. Visitation with the newborn was facilitated. There was no evidence of interval development of decompensated right heart failure in any patient during their postpartum inpatient observation. There was 100% maternal and fetal survival. 

Postpartum, all of the patients were monitored for at least 72 h by our inpatient PH service, which is an intermediate level cardiac unit with telemetry. Daily BNP monitoring along with serial (multiple times daily) assessment of JVP were used to guide diuretic therapy to mitigate right heart congestion. 

Only Patient 1, after pregnancy 1, successfully breast fed. Patient preference, use of diuretics or resumption of ERA therapy otherwise precluded breast feeding. Contraception counseling and family planning were discussed before delivery planning and before the patients were discharged. All patients were followed up in the outpatient PH Program 1–2 weeks after delivery, with ongoing clinical surveillance and reinstitution or escalation of PH medical therapy as warranted to maintain low risk status. 

In the cohort of patients with PAH and pregnancy at our single PH center from 2013 to 2021, there were also three patients that underwent medical pregnancy termination. Two were based on high-risk PAH features, as described herein. The third was based on teratogenic risk of exposure to warfarin when combined with the general estimated risk of maternal and fetal morbidity and mortality in pregnancy in PAH. 

The first patient was 22 years old with hereditary PAH and on high dose combination therapy including subcutaneous treprostinil 108 ng/kg/min, macitentan, sildenafil 80 mg three times daily, and imatinib. She was being evaluated for lung transplantation for ongoing high risk PAH features at the time she became pregnant. In the short term that she held macitentan and imatinib in early pregnancy due to teratogenic risk, her clinical status further declined with severe worsening RV dilation (from mild to severe) and dysfunction (TAPSE from 2.2 cm to 1.3 cm), a three-fold increase in BNP, and worsening 6MWD by 129 m. She underwent dilation and curettage at 9 weeks gestation without complication and resumed PAH medical therapy with clinical improvement thereafter.

The second patient was 38 years old and admitted to the hospital with a new diagnosis of PAH and pregnancy. She has several high-risk features, including severe clinical right heart failure with anasarca and JVP > 20 cm H_2_O, rapid rate of symptom progression, NYHA FC IV, cardiac index 2.0 L/min/m^2^, right atrial pressure 15 mmHg, pericardial effusion and severe RV enlargement and dysfunction. She was nonadherent to medical therapy and medical appointments, and unfortunately suffered from an ongoing substance abuse disorder. Due to severe PAH high risk features early in pregnancy, she elected to undergo dilation and curettage at 8 weeks gestation, without complication. 

The third patient was 30 years old with congenital heart disease associated PAH. She was on sildenafil and warfarin and became pregnant before her first evaluation in our PH center. She had mild RV dilation and dysfunction, JVP 5 cm H_2_O, and BNP 34 pg/mL. She did not have high risk clinical features of PAH, but after consultation with MFM and genetics counseling, she chose to terminate pregnancy due to the teratogenic risk of warfarin exposure, including structural malformations of bone and cartilage such as nasal hypoplasia, stippled epiphyses, and limb hypoplasia, along with central nervous systemic, ophthalmic anomalies, hearing loss, and intrauterine growth retardation risk. She underwent dilation and evacuation at 9 weeks gestation without complication.

## 4. Discussion

In this case series, we report on the clinical, echocardiographic and hemodynamic findings, and management strategies in seven pregnancies of six women with PAH. In all six women and seven deliveries, mother and baby survived and are doing well today. In these women, PAH medical therapy was adjusted either in anticipation of pregnancy or during pregnancy with the intent to optimize right heart function as much as possible. In doing so, all patients achieved low-risk PAH status before delivery. In addition, we report on three patients who were managed with pregnancy termination, due to either a high- risk pathophysiologic state where low-risk status was not attainable or exposure to teratogenic medications.

Normal physiological changes during pregnancy include an increase in blood volume and cardiac output [10,11]. There is a concurrent decrease in the systemic and pulmonary vascular resistance. The increase in cardiac output is achieved by both an increase in heart rate and an increase in stroke volume. By the third trimester, the blood volume has increased by 40% over baseline. In addition, there is a relative hypercoagulable state.

In patients with PAH, the increased right heart afterload limits the ability of intravascular volume expansion and falling systemic vascular resistance to recruit cardiac stroke volume, while increasing the propensity for rising right sided cardiac filling pressures. As pregnancy progresses, functional class and 6MWD may be limited, independent of PAH or RV function, thus limiting interpretability of these parameters. As such, BNP or N-terminal-pro-BNP are important biomarkers that objectively represent cardiac strain and heart failure and have been shown to correlate with risk and prognosis in PAH. Without treatment, right heart dilatation occurs, and in turn, often an increasing degree of TR, leftward interatrial and interventricular septal displacement, and reciprocal reductions in left atrial and left ventricular cavity size. The net result can be falling cardiac stroke volume, cardiac output, rising central venous pressure and hepatic and renal venous congestion. During labor and delivery, there is a further increase in cardiac output related to auto-transfusion and increased blood volume from uterus contraction, and increased sympathetic nervous system activation. Bleeding may lead to relatively rapid reductions in cardiac preload. In addition, there may be associated acidosis and hypercarbia which results in increase in PVR. In PAH, the high-resistance, low-compliance pulmonary circulation and impaired right heart systolic and filling reserve put the patient are increasing risk of right heart failure and total circulatory decompensation at the time of labor and delivery.

These findings together, lend to the high risk of morbidity and mortality during pregnancy for the mother and the fetus [1,4]. When patients of child-bearing age are diagnosed with PAH, there is an emphasis on contraception and prevention of pregnancy. The European Registry on Pregnancy and Cardiac Disease has identified PH as a predictor of heart failure in pregnancy, and has reported maternal death up to 43% in idiopathic PAH [2,5]. Due to these risks, pregnant patients with PAH are often advised to terminate pregnancy. However, pregnancy still occurs in PAH patients and termination may not be an option based on maternal preference or gestational age at presentation. Moreover, pregnancy termination may not be appropriate for PAH patients possessing disease characteristics before pregnancy or at the time of presentation during pregnancy that lend toward effective PAH management and a favorable outcome for mother and baby.

The primary goal of PAH management is to reduce the PVR sufficiently to return right ventricular size and function to a normal or near normal range. In recent work by D’Alto et al. [12] right heart reverse remodeling and functional recovery occurred as a sigmoid function of decreased PVR, with a PVR reduction of 60% or more from baseline leading to dramatic reductions in RV size and normalization of RV systolic function (in turn, greater PVR reduction and improvements in RV size and function are linked to markedly improved WHO functional class, submaximal exercise capacity, and achieving low-risk clinical status [12,13]).

It is logical that the management principles of PAH in general also apply to the management of PAH during pregnancy. Medical therapy should be titrated to reduce the PVR, with the specific intent of achieving normal or near-normal RV size and function. As such, the physiologic cardiovascular adaptations of pregnancy are more likely to occur without right heart and circulatory decompensation and the patient is more able to withstand the physiologic stresses inherent to pregnancy, labor, and delivery with a wider margin of safety. Taken into consideration in medical management must also be the fetal risk of medical therapy (Table 7).

In our patient series, Patients 1, 2, 4, and 5 had known PAH before pregnancy. Thus, all four were on PAH medical therapy before becoming pregnant. Patient 1 was taking an endothelin receptor antagonist (ERA), phosphodiesterase-5 inhibitor (PDE5i) and inhalation treprostinil at baseline, Patient 2 was taking subcutaneous treprostinil therapy, Patient 4 PDE5i oral monotherapy, and Patient 5 combination PDE5i and ERA therapy. Patients 1 and 5 had their ERA stopped before pregnancy, given the known teratogenicity of this class of therapy. Patient 1 had increased dose of inhaled treprostinil and Patient 5 had inhalational treprostinil added to their regimen after the ERA was stopped. All four had a TAPSE of ≥1.8 cm at baseline, with either mild or no TR. In keeping with relative right heart compensation, the highest baseline BNP level in these four subjects was 104 pg/mL, with all others having a BNP level <75 pg/mL. 

In contrast, Patients 3 and 6 in our series were diagnosed with PAH in their third trimester of pregnancy, and thus were untreated for PAH to that point. Patient 3 presented with severe, decompensated right heart failure, severe RV dysfunction and severe TR. This patient was treated with intravenous treprostinil and a PDE5i starting at 28 weeks gestation, and by 34 weeks TAPSE had increased from 1.3 cm to 1.8 cm and the degree of TR had fallen from severe to none. In contrast, Patient 6 presented with only mild RV dysfunction and mild TR in spite of no baseline PAH medical therapy. 

In our patient series, echocardiographic reassessment at a mean of 34 weeks gestation revealed that in six of the seven pregnancies, patients had either mild RV systolic dysfunction or normal RV systolic function combined with either mild or no TR just before delivery. The favorable outcome in our PAH patients during pregnancy mirrors the observation of Ghio et al. [15] who showed that in non-pregnant PAH patients, a TAPSE >1.7 cm combined with grade 0–1+ TR have markedly better survival than subjects with a TAPSE of 1.7 cm or less combined with ≥2+TR. Similarly, in six of the seven pregnancies, there was either no RVOT Doppler notching or only late Doppler notching, consistent with a lower PVR just before delivery [16]. All patients reached low-risk PAH clinical status by the time of delivery. This finding is consistent with recent studies showing a strong relationship between lower PVR, improved right heart function and low-risk clinical status in non-pregnant PAH patients [12,13]. (Taken together, these findings support the notion that the pillars of PAH management including PVR reduction, right heart functional improvement, achievement low-risk clinical status and their relationship to optimal PAH patient survival readily translate as the blueprint for PAH management in pregnancy. 

Jais et al. [17] reported 26 pregnancies in women with PAH. Among the women who did not have a planned or spontaneous abortion and carried pregnancy to delivery, 16 of 20 had a successful delivery and survived without transplantation (those who had a successful pregnancy and delivery had a much lower PVR (6.3 WU) and far better RV function (CI 3.2 L/min/m^2^, RAP 4 mmHg) than the four subjects who died or required transplantation (PVR 20 WU, CI 2.0 L/min/m^2^, RAP 11 mmHg). Interestingly, eight of the sixteen women with successful pregnancies were reported to be long term responders to calcium channel blockers, with an average mPAP of only 30 mmHg and PVR of 4.8 WU. Corbach et al. [18] reported on five women and seven pregnancies, and similar to our cohort, all pregnancies were successful. Like Jais et al., they observed a milder PAH phenotype in their successful pregnant cohort, with an average PVR of 3.7 WU and CI of 3.7 L/min/m^2^. In both studies, oral calcium channel blocker use was highly prevalent, while combination PAH medical therapy and prostanoid use was uncommon.

In our case series, the average baseline mPAP was 49 mmHg and PVR 9.7 WU. In their studies and ours, successful PAH pregnancies were strongly linked to a lower PVR, relatively normal RV function, and thus low-risk PAH status at the time of delivery. However, in our case series, more severe baseline PAH warranted more intensive PAH medical therapy in terms of prostanoid use and combination PAH medical therapy in order to improve RV-PA coupling, low-risk clinical status and a successful pregnancy. 

Yang et al. [6] reported on 7 patients and 7 pregnancies, with 5 of the 7 having known PAH before pregnancy. The average mPAP was 59 mmHg and PVR 11.4 WU. Two of the seven patients died following delivery, and these patients had PVR values of 7.4 and 16.6 WU during pregnancy, suggesting a relative lack of significant hemodynamic improvement during pregnancy. All patients in this cohort received a prostanoid, five of the seven received intravenous epoprostenol, and most received a PDE5i in combination with prostanoid therapy. The two patients died despite veno-arterial extracorporeal membrane oxygenation (VA-ECMO). 

In our cohort, the PAH medical regimen varied significantly among the patients, reflecting an individualized approach, where the intensity of the PAH therapy was chosen based on what was deemed necessary to optimize right heart function and provide sufficient physiologic reserve to ensure an uneventful pregnancy and delivery. We did not ascribe to the use of specific classes or modality of therapy, such as compulsory use of parenteral prostacyclin therapy, for optimal outcome. Reflexive use of prostacyclin therapy and indiscriminate titration of this class of therapy may lead to unintended consequences of excess systemic vasodilation, hypotension, gastrointestinal losses, inappropriately high cardiac output and prostanoid related side effects including but not limited to nausea, diarrhea, thrombocytopenia, flushing, and headache that can adversely affect the health of mother and fetus. Thrombocytopenia may additionally impede the ability to provide neuraxial anesthesia at the time of delivery. We did not employ an inhalational pulmonary vasodilator at the time of 5 of the 7 deliveries, given the patients were thought to be relatively optimized and would not afford any clinical advantage by this approach.

Consideration of VA-ECMO and early involvement of cardiothoracic surgery has been incorporated in the algorithm in high risk PAH patients [19,20,21]. However, in our view, prescriptive use of VA-ECMO at the time of delivery should be strongly avoided, as the complications associated with VA-ECMO in PAH and pregnancy will often far outweigh their benefits. No patients in our cohort required ECMO support. Emphasis should be placed on optimizing right heart function and physiologic reserve through pregnancy and going into delivery, such that the actual need for VA-ECMO should be minimal. The use of VA-ECMO should be reserved for the most unstable patient, in whom standard management has failed. 

In our six patients and seven pregnancies with 100% maternal and fetal survival, we did not place a single invasive PA catheter at the time of delivery. In our center, PA catheter placement is avoided given moment to moment PA pressure monitoring does not correlate with RV performance and in our experience can lead to the use of vasoactive therapy (inotropes or prostacyclin) that may be more detrimental than helpful. If we employed invasive hemodynamic monitoring, a central line was used for central venous pressure monitoring as well as serial assessment of central venous oxygenation (CVO2) saturations for cardiac output assessment. In most cases, clinical assessment of RV compensation was done with serial, and frequent bedside physical examination including JVP, daily BNP monitoring, and repeat echocardiography to guide management through delivery and the postpartum period. Telemetry monitoring was used for arrhythmia assessment, given the maintenance of atrio-ventricular synchrony with normal sinus rhythm in PAH has been shown to contribute markedly to overall RV performance and loss of sinus rhythm would prompt immediate attempts to restore normal sinus rhythm [22].

**Figure 2:**
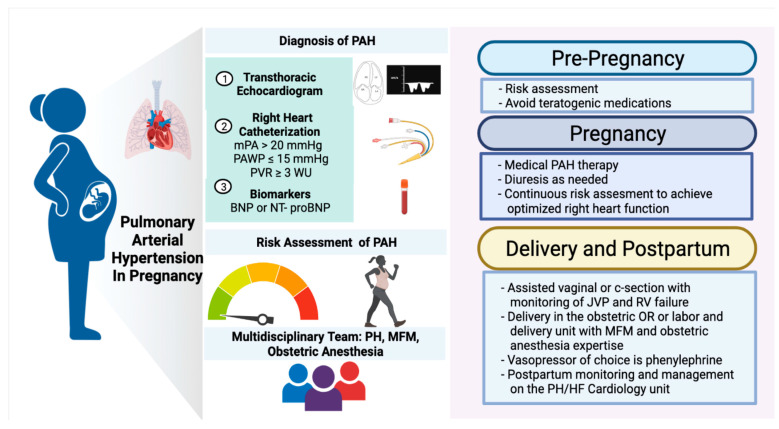
Algorithm Management of PAH during pregnancy. Figure created with Biorender. mPA = mean pulmonary artery; PAWP = pulmonary artery wedge pressure; PVR = pulmonary vascular resistance; WU = Wood units; BNP = brain natriuretic peptide; NT-proBNP = N-terminal pro brain natriuretic peptide; PAH = pulmonary arterial hypertension; JVP = jugular venous pressure; RV = right ventricular; OR = operating room; MFM = maternal fetal medicine; PH = pulmonary hypertension; HF = heart failure.

We did employ a multidisciplinary discussion (Figure 2) involving PH cardiology, maternal fetal medicine (MFM), obstetrics anesthesia, labor and delivery nursing, pharmacy, and if warranted, pediatrics-neonatology, in each pregnancy early in the third trimester to establish the delivery plan. This included a discussion of the anticipated mode of delivery as well as the location of the planned delivery within the hospital setting. In our experience, the mode of anticipated delivery (cesarean or vaginal) should be planned based primarily on obstetric indications and not guided by the presence of PAH. While cesarean delivery may be considered more controlled clinically, it may come with increased risks of bleeding, volume shifts, infection, greater hemodynamic effects of anesthesia, postpartum need for pain control, and limited mobility. 

A guiding principle in our program is to choose the hospital location and staff most experienced with labor and delivery and bring additional resources there if warranted. As such, anesthesiology with obstetrics expertise, rather than cardiac anesthesiology, was preferred. Similarly, delivery occurred in the usual labor and delivery unit or obstetrics OR, rather than in the cardiac ICU or OR. Our PH cardiologists are always present at the time of labor and delivery, to guide PAH specific monitoring and to help mobilize any urgent additional resources if needed in these cases. Thus, allowing the MFM, obstetrics, and anesthesiology teams to focus on the obstetric care and a swift and safe delivery (Figure 2).

In patients undergoing vaginal delivery, the associated volume changes and increased adrenergic stimulation with pain (i.e., tachycardia or arrhythmia) can either precipitate right heart dysfunction or dysrhythmia. Therefore, we did accelerate active labor with oxytocin and the use of vacuum or forceps-assisted delivery as well epidural anesthesia for pain management. 

After delivery, all of our patients were monitored for at least 72 h by our inpatient PH service, with daily BNP monitoring, along with serial JVP assessment, monitoring for interval development of right heart congestion. Close outpatient follow up was arranged for clinical surveillance and reinstitution or escalation of PH medical therapy as warranted to maintain low risk status.

Breast feeding is sometimes encouraged, but data supporting this are limited, and some recommend against this [6,14,23]. If the mother warrants diuretic, this may affect the milk supply [14]. The impact of PAH medical therapy on breast milk is not well studied. There have been published experiences of patients treated with PDE5i and inhaled or parenteral prostacyclin therapy that have breastfed. However, for patients receiving ERA therapy, it is not recommended (Table 7).

We also reported on three patients and three pregnancies that were medically terminated in the first trimester. All subjects underwent pregnancy termination without complication. Termination was advised in the setting of either end-stage PAH, severe disease with a very low prospect of patient adherence to medical therapy and follow up, or undue fetal risk due to teratogen exposure. Our findings are generally in line with previous reports that PAH patients who undergo pregnancy termination have a higher risk profile than those who proceed to delivery [17].

## 5. Conclusions

PAH is a progressive and potentially fatal form of pulmonary vascular disease and afterload-dependent right heart failure. While considered high risk and contraindicated, pregnancy in patients with PAH still does occur. Herein we describe our program’s experience with successful clinical outcomes in patients with PAH. While we are not advocating for pregnancy in our patients with PAH, when it occurs, it must be cared for by an experienced and specialized PH medical team, with a careful baseline PAH assessment followed by individually tailored PH medical therapy to ensure optimized right heart function and physiologic reserve throughout pregnancy and delivery. A multidisciplinary approach with clear planning and communication is paramount to ensure the safest possible outcomes for this high-risk medical condition.

## Figures and Tables

**Table 1:** Clinical characteristics.

	Patient 1		Patient 2	Patient 3	Patient 4	Patient 5	Patient 6
Age (y)	32	37	28	31	24	34	35
PAH Etiology	Secundum ASD status post closure		Idiopathic PAH	Idiopathic PAH	Idiopathic PAH	Unrestricted VSD, Eisenmenger Syndrome	SLE-associated PAH
Comorbidities	Thrombocytopenia		Chronic hypoxia, pre-eclampsia, OSA, thrombocytopenia, morbid obesity		COVID-19	Chronic hypoxia, pre-eclampsia, thrombocytopenia, IUGR	SLE
Gravidity and Parity	G1P0	G2P1001	G1P0	G2P1001	G2P1	G1P0	G2P1
Pre-pregnancy PAH therapy	Ambrisentan 10 mg, sildenafil 60 mg TID and inhaled treprostinil 12 inh/q6h		SC treprostinil 40 ng/kg/min	None	Tadalafil 40 mg daily	Ambrisentan 10 mg (stopped 5 months pre-pregnancy) and sildenafil 60 mg TID	None
Pregnancy PAH therapy	Sildenafil 60 mg TID and inhaled treprostinil 12 inh/q6h		SC treprostinil 40 ng/kg/min and sildenafil 40 mg TID	IV treprostinil 51 ng/kg/min and sildenafil 60 mg TID	Tadalafil 40 mg daily	Inhaled treprostinil 9 inh/q6h and sildenafil 60 mg TID	Tadalafil 40 mg daily and inhaled treprostinil 12 inh/q6
Inhaled epoprostenol 50 ng/kg/min used at the time of delivery	No	No	No	No	Yes	No	Yes
BMI (kg/m^2^)	24	24	56	25	33	18	24
Gestational Age at Delivery	40 wk 3 d	37 wk	31 wk 2 d; Di-Di twins	36 wk 6 d	37 wk	33 wk 3 d	34 wk 5 d
Delivery Indication and Mode	VAVD	VAVD	Pre-eclampsia with severe features; Primary c-section	VAVD	History of prior c-section; Repeat c-section	Pre-eclampsia with severe features; Primary c-section	History of prior c-section; Repeat c-section
Mode of Anesthesia	Epidural	Epidural	Epidural	Epidural	General anesthesia due to hypotension with combined spinal epidural	Epidural	Epidural
Length of Stay Postpartum (days)	3	4	4	4	2	7	11
Maternal Outcome	Alive, Breastfeeding	Alive, RHF postpartum lead her to stop breastfeeding and restart ERA	Alive and well	Alive, decompensated right heart failure with volume overload after delivery requiring diuresis	Alive and well	Alive and well	Alive and well
Neonatal weight (gm)	2898	2823	1441 1385	2370	3034	1561	2450
APGAR Scores	8/9	9/9	4/7/9 8/8	8/9	9/9	9/9	9/9
Contraception method Postpartum	N/A	N/A	IUD	Tubal ligation	IUD	Etonogestrel implant	Tubal ligation

ASD = atrial septal defect; BMI = body mass index; BNP = brain natriuretic peptide; c-section = cesarean section; COVID-19 = coronavirus-19; d = days; Di-Di = dichorionic diamniotic; ERA = endothelin receptor antagonist; FC = functional class; IUD = intra-uterine device; IV = intravenous; meds = medications; OSA = obstructive sleep apnea; PA = pulmonary artery pressure; PAH = pulmonary arterial hypertension; PP = postpartum; PVR = pulmonary vascular resistance; RA = right atrium; RHF = right heart failure; RV = right ventricle; SLE = systemic lupus erythematosus; wk = weeks; WU = Wood units; SC = subcutaneous; VAVD = vacuum assisted vaginal delivery; VSD = ventricular septal defect.

**Table 2:** Functional capacity at diagnosis of pregnancy, BNP at diagnosis of pregnancy and third trimester, and jugular venous pressure at time of delivery.

	Patient 1		Patient 2	Patient 3	Patient 4	Patient 5	Patient 6
WHO/NYHA FC	I	II	II	IV	II	II	III
6MWD (meters)	566	585	N/A	N/A	292	439	357
Baseline BNP (pg/mL)	53	104	3	124	18	58	20
Third trimester BNP (pg/mL)	25	198	13	76	29	69	14
JVP at the time of delivery (cm H_2_O)	6	8	8	10	6	8	8

WHO = World Health Organization; NYHA = New York Heart Association; FC = functional class; 6MWD = six minute walk distance; BNP = B-type natriuetic peptide; JVP = jugular venous pressure.

**Table 3:** Echocardiogram parameters immediately before or first in pregnancy.

	Patient 1		Patient 2	Patient 3	Patient 4	Patient 5	Patient 6
Gestational Age (wk)	18	1 month Pre pregnancy	11	28	4	6	28
RV Enlargement	Mild	Moderate	Mild	Severe	None	Mild	Moderate
RV dysfunction	Mild	Mild	None	Severe	None	Mild	Mild
TAPSE (cm)	1.9	1.8	2.3	1.3	2.5	1.8	1.8
TR	None	Mild	None	Severe	Mild	Mild	Mild
Systolic Flattening	None	Mild	Mild	Severe	None	Moderate	Mild
RVOT pulse wave Doppler notch	None	None	Late	Mid	None	Late	Mid
Pericardial effusion	None	None	None	Moderate	None	None	None
RVSP (mmHg)	N/A	N/A	N/A	100	28	54	70
Pre-pregnancy PH regimen	Ambrisentan 10 mg, sildenafil 60 mg TID and inhaled treprostinil 9 inh/q6h		SC treprostinil 40 ng/kg/min	None	Tadalafil 40 mg daily	Ambrisentan 10 mg (stopped 5 months pre-pregnancy) and sildenafil 60 mg TID	None

N/A = not available due to lack of TR; RVOT = right ventricle outflow tract; RVSP = right ventricular systolic pressure; wk = weeks; SC = subcutaneous; TAPSE = triscupid annular plane systolic excursion; TR = Tricuspid regurgitation.

**Table 4:** Echocardiogram parameters during the third trimester.

	Patient 1		Patient 2	Patient 3	Patient 4	Patient 5	Patient 6
Gestational Age (wk)	37	36	29	34	37	33	34
RV Enlargement	Moderate	Severe	Mild	Moderate	None	Mild	Mild
RV dysfunction	Mild	Moderate	None	Mild	None	Mild	Mild
TAPSE (cm)	1.8	1.7	2.0	1.8	2.9	1.8	1.7
TR	Mild	Moderate	None	None	Mild	Mild	Mild
Septal Flattening	Moderate	Severe	None	Mild	None	Moderate	None
RVOT pulse wave Doppler notch	None	Late	None	None	None	Mid	None
Pericardial effusion	None	None	None	Mild	None	None	None
RVSP (mmHg)	65	75	N/A	50	40	48	53
Medications at the time of the echocardiogram	Sildenafil 60 mg TID and inhaled treprostinil 12 inh/q6h		SC treprostinil 40 ng/kg/min and Sildenafil 40 mg TID	IV treprostinil 51 ng/kg/min and sildenafil 60 mg TID	Tadalafil 40 mg daily	Inhaled treprostinil 9 inh/q6h and sildenafil 60 mg PO TID	Tadalafil 40 mg daily and inhaled treprostinil 12 inh/q6h

N/A = not available due to lack of TR; RVOT = right ventricle outflow tract; RVSP = right ventricular systolic pressure; wk = weeks; SC = subcutaneous; TAPSE = triscupid annular plane systolic excursion; TR = Tricuspid regurgitation.

**Table 5:** Hemodynamic characteristics.

	Patient 1		Patient 2	Patient 3	Patient 4	Patient 5	Patient 6
Gestational Age (wk)	1.5 years before pregnancy	1 year before pregnancy	18	28	9	1 year before pregnancy	29
Medications at the time of RHC	Ambrisentan 10 mg, sildenafil 60 mg TID and inhaled treprostinil 9 (first pregnancy) or 12 (second pregnancy) inh/q6h		SC treprostinil 40 ng/kg/min	None	Tadalafil 40 mg daily	Ambrisentan 10 mg and sildenafil 60 mg TID	None
RA (mmHg)	1	1	10	19	1	4	6
PA (mmHg)	70/30 (43)	60/30 (43)	100/45 (62)	103/31 (63)	20/5 (10) *	100/40 (65)	75/24 (41)
PAWP (mmHg)	8	12	12	7	4	7	14
CO (L/min)	4.4	3.8	5.4	3.3	4.9	2.5	4.4
CI (L/min/m^2^)	2.6	2.3	2.2	1.8	3.3	1.7	2.7
PVR (WU)	7.9	7.2	9.3	16.9	1.4	17	6.1
SVR (dynes-sec-cm^−5^)	1480	1653	1352	1829	1303	2600	1231

RHC = right heart catheterization; RA = right atrial pressure; PA = pulmonary artery pressure; PAWP = pulmonary arterial wedge pressure; CO = cardiac output; CI = cardiac index; PVR = pulmonary vascular resistance; SVR = systemic vascular resistance; wk = weeks; WU = Wood units. * Baseline hemodynamics on patient 4: PA 50/19 (29) mmHg, PAWP 8 mmHg, PVR 3.5 WU.

**Table 6:** PAH Risk Category by ESC/ERS Guidelines.

	Patient 1		Patient 2	Patient 3	Patient 4	Patient 5	Patient 6
At diagnosis of pregnancy	low	low	low	high	low	moderate	moderate
Third trimester	low	low	low	low	low	low	low

ESC = European Society of Cardiology; ERS = European Respiratory Society.

**Table 7:** Fetal risk of medical therapy.

Medication Class		FDA Pregnancy Category	Recommendation during Pregnancy	Recommendation during Lactation
Calcium Channel blocker	Nifedipine		Crosses placenta	Present in breast milk. Limited use. Acceptable when relative infant dose is below 10% [14]
	Diltiazem		Avoid	Avoid
PDE5i	Sildenafil	B	Benefit: avoid HF, stroke, preterm delivery, and maternal/fetal death. Risk: May cause fetal growth restriction.	Present in breast milk.
	Tadalafil	B	data	
ERA	Bosentan		Contraindicated	Unknown
	Ambrisentan	X		
	Macitentan			
Prostacyclin or Prostacyclin analogue	Iloprost	C	Benefit vs. Risk	Unknown, not recommended
	Epoprostenol	Not assigned	Benefit vs. Risk	Unknown, use with caution
	Treprostinil	C (oral), B (inhaled), Not assigned (parental)	Benefit vs. risk	Unknown, use with caution
	Selexipag	Not assigned	Limited data	Not recommended
Guanylate cyclase stimulator	Riociguat	X	Contraindicated	Contraindicated
Digoxin		C		Use with caution
Diuretics		C	Continue	Continue
MRA	Spironolactone Eplerenone	C C	Contraindicated	Avoid
Oxygen			SpO2 above 95%	

ERA = endothelin receptor antagonist; MRA = mineraloid receptor agonist; PDE5i = Phosphodiesterase 5 inhibitor.

## Data Availability

The data are not publicly available due to patient privacy and confidentiality.

## References

[1] Weiss B.M., Zemp L., Seifert B., Hess O.M. (1998). Outcome of pulmonary vascular disease in pregnancy: A systematic overview from 1978 through 1996. J. Am. Coll. Cardiol..

[2] Sliwa K., van Hagen I.M., Budts W., Swan L., Sinagra G., Caruana M., Blanco M.V., Wagenaar L.J., Johnson M.R., Webb G. (2016). Pulmonary hypertension and pregnancy outcomes: Data from the Registry of Pregnancy and Cardiac Disease (ROPAC) of the European Society of Cardiology. Eur. J. Heart Fail..

[3] Luo J., Shi H., Xu L., Su W., Li J. (2020). Pregnancy outcomes in patients with pulmonary arterial hypertension: A retrospective study. Medicine.

[4] Bonnin M., Mercier F.J., Sitbon O., Roger-Christoph S., Jais X., Humbert M., Audibert F., Frydman R., Simonneau G., Benhamou D. (2005). Severe pulmonary hypertension during pregnancy: Mode of delivery and anesthetic management of 15 consecutive cases. Anesthesiology.

[5] van Hagen I.M., Boersma E., Johnson M.R., Thorne S.A., Parsonage W.A., Escribano Subias P., Leśniak-Sobelga A., Irtyuga O., Sorour K.A., Taha N. (2016). Global cardiac risk assessment in the Registry of Pregnancy and Cardiac disease: Results of a registry from the European Society of Cardiology. Eur. J. Heart Fail..

[6] Yang J.Z., Fernandes T.M., Kim N.H., Poch D.S., Kerr K.M., Lombardi S., Melber D., Kelly T., Papamatheakis D.G. (2021). Pregnancy and pulmonary arterial hypertension: A case series and literature review. Am. J. Obstet. Gynecol. MFM.

[7] Regitz-Zagrosek V., Blomstrom Lundqvist C., Borghi C., Cifkova R., Ferreira R., Foidart J.-M., Gibbs J.S.R., European Society of Gynecology, Association for European Paediatric Cardiology, German Society for Gender Medicine (2011). ESC Guidelines on the management of cardiovascular diseases during pregnancy: The Task Force on the Management of Cardiovascular Diseases during Pregnancy of the European Society of Cardiology (ESC). Eur. Heart J..

[8] Bedard E., Dimopoulos K., Gatzoulis M.A. (2009). Has there been any progress made on pregnancy outcomes among women with pulmonary arterial hypertension?. Eur. Heart J..

[9] Opotowsky A.R., Ojeda J., Rogers F., Prasanna V., Clair M., Moko L., Vaidya A., Afilalo J., Forfia P.R. (2012). A simple echocardiographic prediction rule for hemodynamics in pulmonary hypertension. Circ. Cardiovasc. Imaging.

[10] Meah V.L., Cockcroft J.R., Backx K., Shave R., Stohr E.J. (2016). Cardiac output and related haemodynamics during pregnancy: A series of meta-analyses. Heart.

[11] Chapman A.B., Abraham W.T., Zamudio S., Coffin C., Merouani A., Young D., Johnson A., Osorio F., Goldberg C., Moore L.G. (1998). Temporal relationships between hormonal and hemodynamic changes in early human pregnancy. Kidney Int..

[12] D’Alto M., Badagliacca R., Argiento P., Romeo E., Farro A., Papa S., Sarubbi B., Russo M.G., Vizza C.D., Golino P. (2020). Risk Reduction and Right Heart Reverse Remodeling by Upfront Triple Combination Therapy in Pulmonary Arterial Hypertension. Chest.

[13] Maron B.A., Abman S.H., Elliott C.G., Frantz R.P., Hopper R.K., Horn E.M., Nicolls M.R., Shlobin O.A., Shah S.J., Kovacs G. (2021). Pulmonary Arterial Hypertension: Diagnosis, Treatment, and Novel Advances. Am. J. Respir. Crit. Care Med..

[14] Halpern D.G., Weinberg C.R., Pinnelas R., Mehta-Lee S., Economy K.E., Valente A.M. (2019). Use of Medication for Cardiovascular Disease During Pregnancy: JACC State-of-the-Art Review. J. Am. Coll. Cardiol..

[15] Ghio S. (2020). The haemodynamic assessment of patients with pulmonary arterial hypertension. Glob. Cardiol. Sci. Pract..

[16] Arkles J.S., Opotowsky A.R., Ojeda J., Rogers F., Liu T., Prassana V., Marzec L., Palevsky H.I., Ferrari V.A., Forfia P.R. (2011). Shape of the right ventricular Doppler envelope predicts hemodynamics and right heart function in pulmonary hypertension. Am. J. Respir. Crit. Care Med..

[17] Jais X., Olsson K.M., Barbera J.A., Blanco I., Torbicki A., Peacock A., Vizza C.D., Macdonald P., Humbert M., Hoeper M.M. (2012). Pregnancy outcomes in pulmonary arterial hypertension in the modern management era. Eur. Respir. J..

[18] Corbach N., Berlier C., Lichtblau M., Schwarz E.I., Gautschi F., Groth A., Schüpbach R., Krähenmann F., Saxer S., Ulrich S. (2021). Favorable Pregnancy Outcomes in Women with Well-Controlled Pulmonary Arterial Hypertension. Front. Med..

[19] Kamp J.C., von Kaisenberg C., Greve S., Winter L., Park D.H., Fuge J., Kühn C., Hoeper M.M., Olsson K.M. (2021). Pregnancy in pulmonary arterial hypertension: Midterm outcomes of mothers and offspring. J. Heart Lung Transpl..

[20] Ye J., Chen J.Y., Xu N., Wu B., Wang Z.P., Xu H.Y., Ma J.Q. (2019). Bilateral lung transplantation after caesarean section in pregnancy with severe pulmonary arterial hypertension: A case report. Medicine.

[21] Ngatchou W., Ramadan A.S., Van Nooten G., Antoine M. (2012). Left tilt position for easy extracorporeal membrane oxygenation cannula insertion in late pregnancy patients. Interact. Cardiovasc. Thorac. Surg..

[22] Sivak J.A., Raina A., Forfia P.R. (2016). Assessment of the physiologic contribution of right atrial function to total right heart function in patients with and without pulmonary arterial hypertension. Pulm. Circ..

[23] Kiely D.G., Condliffe R., Wilson V.J., Gandhi S.V., Elliot C.A. (2013). Pregnancy and pulmonary hypertension: A practical approach to management. Obstet. Med..

